# Essential Oils and Mono/bi/tri-Metallic Nanocomposites as Alternative Sources of Antimicrobial Agents to Combat Multidrug-Resistant Pathogenic Microorganisms: An Overview

**DOI:** 10.3390/molecules25051058

**Published:** 2020-02-27

**Authors:** Nagaraj Basavegowda, Jayanta Kumar Patra, Kwang-Hyun Baek

**Affiliations:** 1 Department of Biotechnology, Yeungnam University, Gyeongsan, Gyeongbuk 38451, Korea; nagarajb2005@yahoo.co.in; 2 Research Institute of Biotechnology and Medical Converged Science, Dongguk University-Seoul, Goyang 10326, Korea

**Keywords:** essential oil, bi-metallic nanoparticles, tri-metallic nanoparticles, synergistic effect, antimicrobial activities, multidrug-resistant pathogens

## Abstract

Over the past few decades, many pathogenic bacteria have become resistant to existing antibiotics, which has become a threat to infectious disease control worldwide. Hence, there has been an extensive search for new, efficient, and alternative sources of antimicrobial agents to combat multidrug-resistant pathogenic microorganisms. Numerous studies have reported the potential of both essential oils and metal/metal oxide nanocomposites with broad spectra of bioactivities including antioxidant, anticancer, and antimicrobial attributes. However, only monometallic nanoparticles combined with essential oils have been reported on so far with limited data. Bi- and tri-metallic nanoparticles have attracted immense attention because of their diverse sizes, shapes, high surface-to-volume ratios, activities, physical and chemical stability, and greater degree of selectivity. Combination therapy is currently blooming and represents a potential area that requires greater attention and is worthy of future investigations. This review summarizes the synergistic effects of essential oils with other antimicrobial combinations such as mono-, bi-, and tri-metallic nanocomposites. Thus, the various aspects of this comprehensive review may prove useful in the development of new and alternative therapeutics against antibiotic resistant pathogens in the future.

## 1. Introduction

Infectious diseases and foodborne illnesses are the leading cause of severe health problems worldwide and can even lead to death. Antibiotics and other antimicrobial agents are the major strategies used to combat pathogenic bacteria in human medicine [1]. However, inappropriate over prescription and irrational use of antibiotics in the treatment of infectious diseases has led to favorable conditions, exposure, and spread of resistant strains of different pathogens [2]. These negative health trends require urgent attention from scientific institutions and pharmaceutical researchers to develop novel therapeutics with different strategies for the prevention and treatment of infectious diseases [3]. Infectious diseases resulting from multidrug-resistant bacteria have become abundant, especially vancomycin-resistant enterococci, methicillin-resistant *Staphylococcus aureus*, penicillin-resistant *Streptococcus pneumoniae*, ceftazidime-resistant *Klebsiella pneumonia* and *Escherichia coli*, and *Pseudomonas aeruginosa* resistant to fluoroquinolones [4]. In addition, various types of foodborne pathogens associated with Gram-positive and Gram-negative bacteria such as *Bacillus cereus*, *E. coli*, *Listeria monocytogenes*, *Salmonella enteritidis*, and *S. aureus* present a major threat to public health and safety [5]. Owing to these problems, there has been renewed interest in alternative research for more effective, less toxic antimicrobial agents among natural bioactive compounds, which are found in aromatic plants, and have been used in cosmetics, aromatherapy, and folk medicine for many years, such as essential oils and plant extracts [6]. Traditionally, medicinal and aromatic plants have received significant attention for the treatment of human diseases because of their pharmacological activities, low toxicity, and economic viability [7]. The presence of active phytochemicals or bioactive compounds and their secondary metabolites, or essential oils in plants play an important role against the problem of antibiotic resistance in bacteria. Some of these alternative antimicrobial therapies may reduce the further spread and development of resistance in these pathogens. 

Essential oils (EOs) are natural mixtures of volatile secondary metabolites extracted from different parts of plants, such as the flowers, buds, seeds, leaves, twigs, bark, herbs, wood, fruits, and roots [8]. EOs may have received their name because they were the essence of odor and flavor or at one time, they were considered essential to life processes. The most common chemical constituents of EOs include phenols, polyphenols, flavonols, tannins, quinones, terpenoids, flavonoids, flavones, coumarins, alkaloids, lectins, and polypeptides that exhibit potential biological activities such as anti-inflammatory, antioxidant, insecticidal, anti-allergic, antiseptic, antiviral, anti-parasitic, anticancer, and antimicrobial properties [9]. In addition, many plants’ EOs are useful as aroma in aromatherapy, flavor in food and its additives, and enhancers in cosmetics, soaps, plastics, resins, and perfumes [10]. EOs are obtained through various methods such as vacuum distillation, fermentation, solvent extraction, simultaneous distillation, microwave-assisted extraction, supercritical fluid extraction, microwave hydrodistillation, steam distillation, and static, dynamic, and high-concentration capacity headspace sampling methods [11]. The EOs obtained contain a complex mixture of 20–60 bioactive compounds at different concentrations. The yield can vary between different bioactive compounds and may differ among different plant species and plant parts depending on chemically derived compounds such as aromatic, aliphatic, and phenolic acids, and terpenes [12]. The differences in the chemical composition of EOs are due to exogenous and endogenous factors, which may lead to chemotypes or ecotypes. The exogenous factors depend on environmental factors such as light, temperature, weather conditions, precipitation, growing site, and soil. The endogenous factors are associated with anatomical and physiological characteristics of the plants like chemical variation and genetically related factors [13,14].

EOs contain various components which have been screened for their antimicrobial activities. Among these, terpenes—such as carvacrol, geraniol, menthol, and thymol—have higher antimicrobial properties. Compared to the individual components, the whole or multicomponent EOs appear to have greater antibacterial activity. However, each single chemical component in EOs plays an important role, such as in the density, fragrance, color, texture, cell penetration ability, fixation on cell walls, and bioavailability. Therefore, it is assumed that the other molecules present in EOs regulate the function of the main components to enhance synergistic effects [15]. Thus, the combination of antimicrobials with other antimicrobial agents provides many benefits, including higher biological activity, and reduces the adverse effects and toxicity of the combined components. In synergistic activity, one antimicrobial agent enhances the activity of the other, and finally they may act together more effectively [16]. This could be a new approach to solving the problem of bacterial resistance and reduced susceptibility. Most importantly, association of other antimicrobial agents with EOs targeting resistant bacteria may have different mechanisms of action, and it may lead to new choices to overcome the assault of microbial resistance. Various in vitro studies have confirmed the antimicrobial activities of some essential/volatile oils, plant extracts, and antibiotics against different microorganisms using different methods, and the synergistic effects of EOs/EOs, plant extract/plant extract, plant extract/EOs, plant extract/conventional antibiotics, and phytochemical/antibiotics [16], with a significant reduction in minimum inhibitory concentration. 

Nanotechnology has proved to be a powerful tool for solving various biomedical and technological problems using its predefined structures. This field provides the power of transforming the structures of atoms or molecules into desired geometry and properties. It has both health and environmental applications, which includes effective drug delivery, cancer treatment, food packaging, harvesting solar energy, and water purification, and reduces the use of industrial chemicals, thereby making the environment healthier and safer [17]. Over the past few decades, many metal and metal oxide nanoparticles (NPs) have been studied extensively due to their distinctive properties, and their potential applications in various fields such as biomedical, biosensors, catalysis, electronics, optoelectronics, information storage, and surface-enhanced Raman spectroscopy (SERS) [18]. As the name indicates, monometallic NPs contain a single metal, which can be prepared either by biological or chemical methods whereas bi- and tri-metallic NPs are formed by the combination of two or more metals, which exhibit fascinating properties. Compared to monometallic NPs, bi- and tri-metallic NPs have drawn greater interest because of their importance in optical, electrical, and catalytic applications in various fields [19]. Transition bi- and tri-metallic NPs are used as catalysts in many organic reactions and show higher catalytic activities compared to monometallic NPs [20,21,22,23]. Bi-metallic NPs such as FePd, AuPd, PtPd, and CuPd [24,25,26,27], and tri-metallic NPs such as AuFeAg and FeAgPt [19,23] are used as heterogeneous catalysts with excellent selectivity and activity compared to monometallic NPs. It has been proven that it is possible to tune the shape, size, and properties of NPs as heterodimers [28], nano-alloys [23], and core–shell [29] for further catalytic applications. However, only monometallic NPs along with EOs have so far been reported to have limited data to target resistant bacteria. 

**Figure 1:**
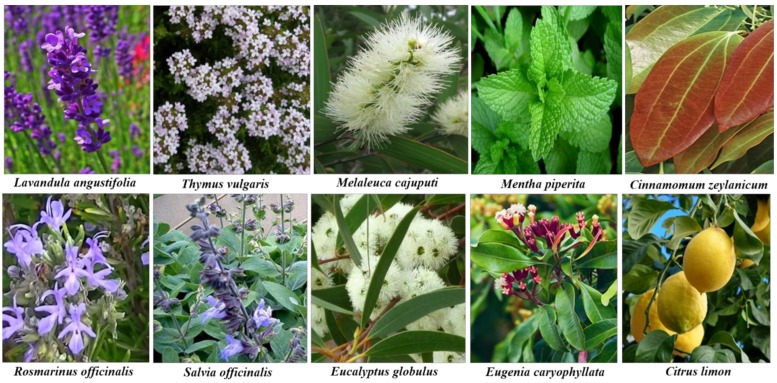
Widely used medicinal plants with high antimicrobial activities against human pathogens.

This review highlights the most recent literature on EOs, with special focus on mono, bi-and tri-metallic NPs, along with their antimicrobial potential. We have discussed the extraction methods, chemical composition, antimicrobial effects of EOs, nano-encapsulation of EOs, and their synergistic effects on infection resistant pathogens. Potential effects and antimicrobial activities of mono-, bi-, and tri-metallic NPs will also be discussed. Additionally, the future prospects of the synergistic effects of EOs and bi- and tri-metallic NPs have also been discussed herein. Some important and widely used medicinal plants act as antimicrobial activities are depicted in Figure 1.

## 2. Methods of Extraction of Essential Oils

EOs can be extracted from different parts of plants using several methods with appropriate solvents and techniques. Based on the characteristics of different plant materials, some specific extraction techniques are used for extracting the volatile fraction from aromatic plants. The techniques include solvent extraction, solvent flavor evaporation, Soxhlet extraction, maceration, CO_2_ extraction, hydrodistillation, steam distillation, dry distillation, mechanical cold pressing, microwave-assisted extraction, supercritical fluid extraction, simultaneous distillation extraction, vacuum distillation, solid-phase microextraction, direct thermal desorption, dynamic headspace, static headspace, and high-concentration capacity headspace sampling [30,31]. The yield and success of extraction depends on the type and length of extraction period, solvent, climate, the plant organ, age, temperature, pH, soil composition, and vegetative cycle stage [32,33,34,35]. Generally, dried plant material and water are used as a solvent for the extraction of EOs. However, some components of EOs are not soluble in water due to low polarity, therefore, to increase the polarity, organic solvents may be used, such as acetone, ether, petroleum ether, ethanol, chloroform, hexane, and ethyl acetate [36,37]. EOs produced using organic solvents are not considered by the National Cancer Institute as the oil residues can alter the quality of the EOs and lead to impurity [38].

Modern methods such as supercritical fluid extraction and microwave-assisted extraction were achieved at the laboratory scale, but they are intuitively difficult to expect a reliable scale-up. Solvent extraction is one of the most convenient and frequently used method to extract bioactive compounds from plant materials. Solvent extraction method is a simple and efficient method used to separate a compound into its components based on the solubility of the components when it is mixed with a solvent. It is important to choose a good solvent since a residue of the solvent could be present in the final product due to its polarity, viscosity, and vapor pressure. High solvent requirement, long extraction period, and unsatisfactory reproducibility are some few disadvantages of this method [39,40].

Hydrodistillation is a simple and traditional method for extracting EOs from plant samples and is further classified into the subcategories of water distillation, steam distillation, and direct steam distillation [41]. In this method, samples are packed into a still compartment, sufficient quantity of water is added, and the mixture is boiled by applying mild heat (water distillation); alternatively, live steam (steam distillation) followed by direct steam (direct steam distillation) can also be injected into the plant material. Hot water and steam act as the main influential factors that liberate EOs from the plant tissues. The vapor, which is a mixture of water and oil, is condensed with cooling water. The condensed mixture flows from the condenser to a separator, where the bioactive compounds and oils are separated from the water [42].

Soxhlet extraction is a common conventional method that involves solid–liquid contact for extracting several compounds. This extraction method uses chemical solvents to extract oils; the solvent is heated in a distillation flask and the resulting vapor is condensed. The condensed solvent from the condenser flows into the thimble that contains the sample. When the solution reaches an overflow, a siphon pulls the solution in the thimble back into the distillation flask, thus carrying dissolved solute into the bulk liquid [39,43]. This procedure should be repeated by washing with an organic solvent until extraction is completed.

Maceration is the simplest process of extraction; the whole or coarsely powdered sample is mixed with solvent and left to macerate for a known period with frequent agitations at room temperature. After maceration, the sample mixture is pressed and filtered through an appropriate filter [39]. Cold pressing method is one of the best methods used to extract oils. In this method, the whole plant is pressed at low temperature and pressure to squeeze the material from the pulp to release the EO. Supercritical fluid extraction is the process of separation of bioactive components using CO_2_ as an ideal solvent. This technique requires low pressure, moderate temperature, and CO_2_ as solvent for a wide variety of applications such as EO and metal cation extraction. CO_2_ is non-toxic, non-explosive, noncorrosive, readily available, safe, inexpensive, and easily eliminated from the extract [44,45].

Ultrasound-assisted extraction provides a cutting edge with higher yields, superior quality, clean process, and less energy. Sonication process was carried out as a solvent- or water-based method to penetrate into the plant cells via bubble implosion generated by ultrasonic cavitation [46]. The bubble implosion creates micro-jets, which pulverize the lipid glands in the plant cell tissue and the process prevents the degradation of extracts [47]. Microwave-assisted extraction technique is more efficient and bio-sustainable, and is an alternative to conventional heating because it reduces extraction time, costs, energy, solvent consumption, and CO_2_ emissions [48,49]. In this method, plant materials are dispersed in solvents, and the mixture exposed to microwaves, then the interaction between the microwave irradiation and solvent releases the EOs [50]. Infusion and decoction are the popular traditional methods for the preparation of aqueous extracts with cold or boiling water for a fixed time duration [51]. Some different plant EOs and its extraction methods are summarized in Table 1.

## 3. Chemical Composition of Essential Oils

The chemical composition of EOs are attributed to various factors, e.g., plant species, climatic conditions, soil type, temperature, humidity, ecotype, phenophase, photoperiod, irradiance, genotype, harvesting seasons, age of leaves, agronomic conditions, geographic region, and extraction process [60,61,62]. EOs are complex mixtures of aromatic and volatile compounds with many single compounds, but the number can vary in different plant materials. Most EOs are composed of aromatic, aliphatic constituents; terpenes, terpenoids with low molecular weights; lipophilic, highly volatile, secondary plant metabolites; mono- and sesquiterpenes; and allyl and isoallyl phenols. Terpenes are formed by condensation of two or more isoprene units represented by the chemical formula (C_5_H_8_)_n_ through the mevalonic acid pathway, which occurs in the cytoplasm of the cell [63]. Based on the number of carbon atoms present in the structure, terpenes are classified as mono-, sesqui-, di-, ses-, tri-, and tetra-terpenes (carotenoids), and alternative hemi-terpenes. Many terpenes are hydrocarbons, but alcohols, aldehydes, or ketones are oxygen-containing compounds and these terpenes are known as terpenoids. Monoterpenes are the most delegate structures composed of two isoprene units covering a wide range of oxidation states like monocyclic, bicyclic, and acyclic forms, and organic functional groups including hydrocarbons (myrcene, camphene, 𝛼-pinene, 𝛼-terpinene, and p-cimene) and alcohols (menthol, nerol, borneol, and linalool). There are other functional groups like aldehydes (geranial and citronellal), esters (citronellyl acetate, linalyl acetate, and menthyl), ketones (camphor, pulegone, and carvone), peroxides (ascaridole), phenols (carvacrol and thymol), and ethers (1,8-cineole and menthofurane), which are also main constituents of EOs [64]. Sesquiterpenes are major types of terpenes formed from the combination of three isoprene units. Sesquiterpenes provide the spicy note, and are unsaturated compounds, which include hydrocarbons (azulene, 𝛽-bisabolene, cadinenes, germacrene D, humulene, farnesenes, zingiberene, and 𝛽-caryophyllene), oxygenated sesquiterpenes (caryophyllene oxide, spathulenol, and nerolidol), alcohols (patchoulol, bisabolol, 𝛽-nerolidol, farnesol, 𝛽-santalol, and patchoulol), acids (benzoic acid and geranic acid), aldehydes (citral), ketones (germacrone, benzophenone, acetophenone, 𝛽-vetinone, and turmerones), epoxides (caryophyllene oxide and humulene epoxides), and lactones (bergapten) [65,66].

**Figure 2:**
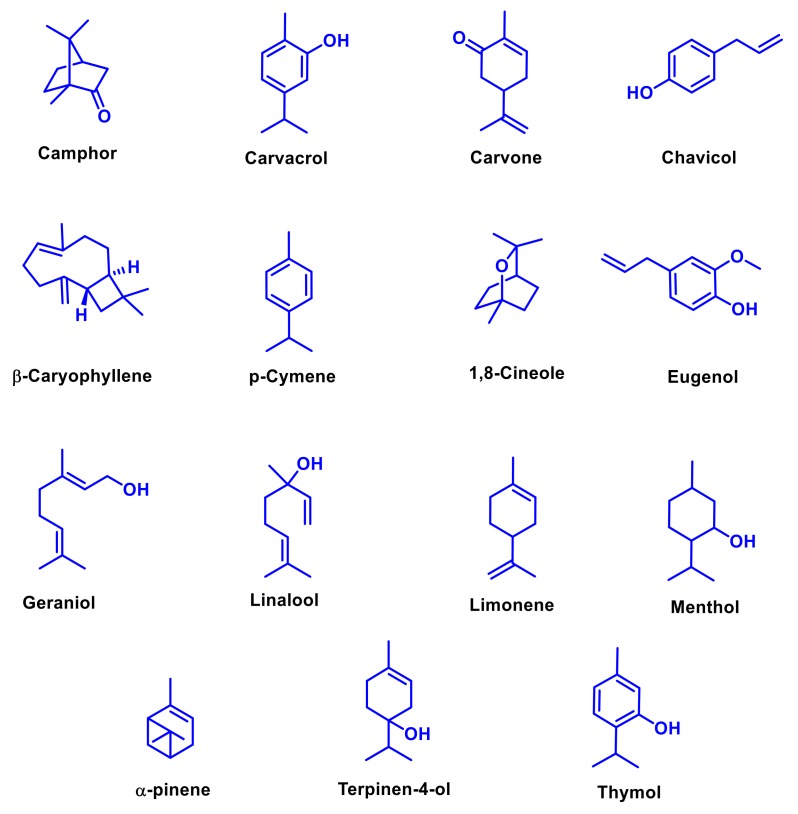
Major and biologically important bioactive constituents present in essential oils.

Some different kinds of organic compounds—such as sterols [67], alkaloids [68], tannins [69], and flavonoids [70]—are also present in EOs. Aromatic compounds occur as relatively small part of EOs when compared to terpenes. They contain alcohol (cinnamyl alcohol); benzene (styrene); aldehydes (cynnamaldehyde); phenols (chavicol, eugenol, vaniline, and cinnamaldehyde); methoxy compounds (methyleugenol, elemicine, estragole, and anethole), and methylene dioxy derivatives (safrole, myristine, and apiole) [71]. The other compounds like nitrogen and sulfur-containing compounds are also present as aglycones or glucosinolates in EOs [72]. The sulfur-containing compounds—namely dimethyl sulfide, allyl sulfide, diallyl sulfide, and dimethylthiophene—are mainly responsible for the characteristic odor and taste [73]. Nitrogen-containing compounds like indole, pyridine, methyl anthranilate, and pyrazine, are found in only a few EOs [74]. The major and biologically important chemical constituents of EOs and its structures are depicted in Figure 2.

## 4. Antimicrobial Effects of Essential Oils

At present, many new antimicrobial agents or antibiotics have been developed from various sources for treating different microbial pathogens. However, the increased use of antibiotics has resulted in the emergence of multidrug resistance bacteria, which has led to the severity of diseases caused by bacterial pathogens. Some of the main multidrug resistance bacteria are *E. coli*, *S. aureus*, *P. aeruginosa*, *Enterococcus* spp., *Salmonella* spp., and coagulase-negative *Staphylococcus*, and are included in the category of community and hospital acquired pathogens, which affects public health [12].

Natural resources like plant extracts widely used as medicinal plants with high antimicrobial activities against human pathogens (Figure 1). EOs and their major bioactive constituents are potential candidates for antibacterial, antifungal, antiviral, antiseptic, antioxidant, anti-parasitic, and insecticidal agents for promoting food preservation, and as alternatives for treating infectious diseases (Figure 2) [75,76]. EOs exhibit wide-ranging inhibitory activities against various bacterial pathogens [77] by easily penetrating the lipids of the bacterial cell membrane and disrupting their cell wall structure [78]. Association of EOs constituents with lipids causes loss of integrity and cellular contents, and finally leads to bacterial cell death [79]. Some of the constituents of EOs such as carvone, a member of terpenoids, split in the lipid membrane, while terpinen-4-ol, an isomer of terpineol, prevent cellular respiration, and both destroy the function of the cell membrane as a permeable barrier [80,81]. Several bioassays are well known and commonly used, such as well diffusion, disk-diffusion, and agar dilution methods, but others such as bioluminescent and flow cytofluorometric methods are not widely used because they require selected equipment [82].

The minimum inhibitory concentration (MIC) is the lowest concentration of antimicrobial agent that completely inhibits growth of the organism in micro-dilution wells or tubes as detected by the unaided eye [83]. However, the determination of minimum bactericidal concentration (MBC) or minimum fungicidal concentration (MFC), also known as minimum lethal concentration (MLC), is the most common estimation of bactericidal or fungicidal activity, which is defined as the lowest concentration of antimicrobial agent needed to kill 99.9% of the final inoculum [84].

The various bioactive components present in different EOs play an individual role; for instance, the EOs of cinnamon and black pepper damaged the cell membrane and decreased the metabolic activity of *E. coli* and *S. aureus* [85,86]. Similarly, EO from *Dipterocarpus gracilis* inhibited the growth of *P. mirabilis* and *B. cereus* by infecting cytoplasmic membrane. The differences in antimicrobial activity of EOs may be associated with the chemical constituents, geographical location, seasons, and extraction methods [87]. Some important plants, which are the most-active EOs and their antimicrobial activity against pathogenic microorganisms, are summarized in Table 2.

## 5. Antioxidant Activity of Essential Oils

Free radicals play an important role in origin of life and biological evolution, leaving beneficial effects on the organisms and involved in many biochemical activities of cells such as signal transduction, gene transcription, and regulation. The human body produces oxygen free radicals and other reactive oxygen species (ROS) as byproducts through several physiological and biochemical processes. However, over production of free radicals can cause oxidative damage to biomolecules leading to many chronic diseases such as cancer, cardiovascular, diabetics, chronic inflammation, and atherosclerosis in humans. Therefore, much attention has been focused on the use of antioxidants to inhibit lipid peroxidation due to free radicals by using synthetics or natural antioxidants. Synthetic antioxidants such as butylated hydroxyanisole (BHA) and butylated hydroxytoluene (BHT) are suspected to be harmful to human health. On the other hand, natural products present in medicinal plants like EOs shows significant antioxidants performance from different plant source. The EOs obtained from *Zanthoxylum alatum*, *Ammodaucus leucotrichus*, *Marrubium globosum*, *Citrus sinensis* and *Citrus latifolia*, *Lawsonia inermis*, *Thymus fontanesii*, *Artemisia herba-alba* and *Rosmarinus officinalis*, *Syzygium aromaticum* L., *Origanum vulgare* L., *Mentha spicata* L., and *Eremanthus erythropappus* M showed promising antioxidant activity [132,133,134,135,136,137].

## 6. Potential Impacts of Bi-Metallic and Tri-Metallic Nanoparticles

Over the past decades, several mono-, bi- and tri-metallic NPs have drawn significant attention due to their catalytic, optical, and magnetic properties in a wide array of fields such as catalysis, medical, imaging, remote sensing, environmental, and energy applications. As the name suggests, monometallic NPs are composed of only a single metal (Au, Pt, or Pd), whereas, bi- and tri-metallic NPs are composed of two and three different metals (Au/Ag, Pt/Pd, Fe/Ag/Pt, and Au/Fe/Ag), respectively. Bi- and tri-metallic NPs have tunable and better properties due to the addition of a second and third metal of the nanoparticle combination, and can improve catalytic activity and selectivity when compared to monometallic NPs [138]. The catalytic activity of the external metal (secondary metal) may increase by the bi-metallization of NPs. 

The properties of bimetallic NPs include electronic, catalytic, thermal, size, and shape, which may differ from those of the monometallic NPs. In tri-metallic NPs, the addition of a third metal modifies the electronic structure, reduces the lattice, and increases the charge shift, catalytic performance, and selectivity when compared to monometallic and bimetallic NPs [139,140]. The preparation conditions regulate the structure, size, and shape of the particles such as alloy, core–shell, and heterodimer of two or more metals in bi- and tri-metallic NPs [23,28,29]. The catalytic performance of mono-, bi-, and tri-metallic NPs was also investigated individually and in combination. However, in our previous reports, bi- and tri-metallic NPs showed superior catalytic activity when compared with monometallic NPs [23]. When one metal alloy is combined with other metals to form bi or tri-metallic NPs, the catalytic properties of the consequent material become better when compared to pure metals. 

Bi- and tri-metallic NPs contain a few tens to several thousand of atoms, which are excellent catalysts, with improved selectivity and efficiency due to their highly active surfaces, and act as green catalysts by recyclability [17]. Multi-metallic structures like bi- and tri-metallic NPs furnish many active inter-metallic interfaces to change the electronic structure [141] and allow tuning of the catalytic activity via composition ratios. So far, bi- and tri-metallic NPs interfaces are more active because of fast electron interchange and the presence of crystal defects, which assures substantial implementation [142]. Several different methods are used to prepare bi- and tri-metallic NPs in the required size, composition, and shape, which influence the properties of the material, including electrochemical reduction, microwave, microemulsion, co-precipitation, pyrolysis, hydrothermal, selective catalytic reduction, sol–gel, solvothermal processes, and combustion [143,144,145,146,147,148,149,150,151,152].

## 7. Antimicrobial Activities of Metallic Nanoparticles

Bacterial resistance has become a severe problem due to the substantial application of antibiotics preferred for the treatment of infectious disease without proper medical indications. In order to solve this problem, the use of alternative antibacterial agents to treat infectious diseases have attracted great interest, due to their heat resistance, sustainability, and improved stability under harsh processing conditions. Due to their smaller dimensions and large surface area to volume ratios, metallic NPs provide strong, targeted, and extended antimicrobial interaction with bacteria and biofilms at smaller doses. In the last few decades, new and novel antimicrobial agents—such as metallic NPs and macromolecules—have been found to be the most effective agents to combat pathogenic bacteria. 

Some metal NPs such as silver (Ag), gold (Au), gallium (Ga), copper (Cu), zinc (Zn), iron (Fe), and palladium (Pd) have potential antimicrobial activities. Metal oxide NPs like aluminum oxide (Al_2_O_3_), iron oxide (Fe_3_O_4_), titanium dioxide (TiO_2_), copper oxide (CuO), zinc oxide (ZnO), cobalt oxide (Co_3_O_4_), manganese oxide (Mn_2_O_3_), magnesium oxide (MgO), indium oxide (In_2_O_3_), silicon dioxide (SiO_2_), nickel oxide (Ni_2_O_3_), zirconium dioxide (ZrO_2_), and chromium oxide (Cr_2_O_3_) have also shown potential antimicrobial activities (Table 3). Metal and metal oxide-based NPs damage the cell membrane by binding and releasing metallic ions into proteins and enzymes of the bacterial cell wall. NPs can attack the bacterial cell wall through several modes of action like electrostatic attraction, van der Waals forces, and hydrophobic interactions [153,154,155]. However, different types of NPs have different mechanisms to combat bacteria by forming pores on the surface of bacterial cell membrane, which in turn causes radical formation, generates reactive oxygen species, inhibits enzyme activity, deactivates proteins and DNA, induces oxidative stress, and modifies gene expression levels [156,157]. 

Higher prevention of bacteria is attained by a large surface area-to-mass ratio, zeta potential, surface morphology, crystal structure, smaller and tunable size and shape of particles, which allows close interaction with microbial membranes. Moreover, other factors that influence antibacterial effects of nanoparticles include bacterial strain, environmental conditions, and the exposure time. Metal and metal oxide nanoparticles were studied for their well-known antimicrobial activities against Gram-positive bacteria like *S. aureus* and *B. subtilis*, and Gram-negative bacteria like *P. aeruginosa* and *E. coli* including antibiotic-resistant strains. In particular, silver NPs alone or in combination with other nanomaterials have great potential antimicrobial activities owing to their unique chemical stability, catalytic activity, and better contact with microorganisms. Similarly, gold NPs also exhibit significant effects of antimicrobial activity but the chemicals used as precursor are expensive. In addition, metal oxide nanoparticles—such as ZnO, CuO, TiO_2_, Al_2_O_3_, and Fe_2_O_3_—have also demonstrated significant effects of antimicrobial activities against both Gram-positive and Gram-negative bacteria. 

## 8. Efficiency of Nano-Encapsulated Essential Oils

It is well known that EOs have shown excellent antimicrobial activity against pathogenic microorganisms. However, their utilization is very limited because of low water solubility and their high sensitivity to oxygen, moisture, heat, and light. To increase their stability, water solubility, and protect EOs from degradation, many modification technologies have emerged as solutions to these existing challenges. Encapsulation of essential oils into nano-based delivery systems such as nanoemulsions, microemulsions, solid-lipid nanoparticles, and liposomes are models for the encapsulation of natural bioactive compounds to improve antimicrobial activities. Currently, the application of nanoencapsulation technology has increased rapidly in the food industry, especially in the EOs industry due to its interesting parameters such as size, zeta potential, and the polydispersity index. 

Nanoemulsions are colloidal dispersions consisting of two immiscible solvents, oil (globules) and water (liquid), in which one is dispersed in the other with the help of a surfactant that stabilizes emulsions. Surfactants are required to formulate nanoemulsions and decrease the size of the droplet and increment the inflexibility and quality of interfacial layer. The combination of Span 20 with Tween 40 was seen to be sophisticated by delivering ideal mineral oil emulsions. For instance, the clove, cinnamon, and thyme oil nanoemulsions which were formulated with nonionic surfactants (Spans and Tweens) were having droplet size less than 100nm. Nanoemulsions are a promising nanocarrier widely used in drug delivery, which helps in improving biodistribution of drugs and minimizing toxicity. To protect EOs from extrinsic and intrinsic factors such as pH, temperature, water, humidity, activity, storage environment, and enzymatic degradation, some different delivery systems have been used as carriers including chitosan cyclodextrin, alginate, albumin, globulin, maltodextrin, and starch. Among the many nanocarriers, naturally occurring polymers such as chitosan and alginate are widely used in the biomedical and pharmaceutical fields as emulsions. 

Many studies in academia and industry have continued to enhance the physical stability of EOs by encapsulating them into nanocarrriers. Recently, nanoemulsions were achieved by using low concentrations of EOs, pectin, and surfactants with the help of pseudo-ternary diagrams [179]. Similarly, nanoencapsulation of lime EOs with chitosan showed enhanced antibacterial activity against *S. aureus*, *L. monocytogenes*, *Shigella dysenteriae*, and *E. coli* as nanoemulsion [180]. Soybean oil with sodium dodecyl sulfate against *S. aureus* as nanoemulsion [181], *Schinus molle* with chitosan against *Aspergillus parasiticus* as nanoprecipitation [182], *Zataria multiflora* with lipid phase (glyceryl monostearat) and precirol against *Aspergillus ochraceus*, *Aspergillus niger*, *Aspergillus flavus*, *Alternaria solani*, *Rhizoctonia solani*, and *Rhizopus stolonifer* as solid-lipid nanoparticles [183]. In addition, *Gaultheria procumbens* with chitosan-cinnamic acid microgel against *A. flavus* as microencapsulation [184], *Thymus capitatus* with sodium dodecyl sulfate against *E. coli* and *B. subtilis* as nanoencapsulation [185], cardamom EO with chitosan against *E. coli* and *S. aureus* as nanocomposites [186], and *Siparuna guianensis* with chitosan against larvicide *Aedes aegypti* as nanoencapsulation [187].

## 9. Interaction of Essential Oils and Metallic Nanoparticles

Nanotechnology emerged significantly in pharmaceutical industry up to a considerable extent. Nanoparticles is a combined name for any colloidal carrier like nanospheres and nanocapsules. EOs can be enhanced by encapsulating with several nanomaterials such as polymeric NPs, liposomes, solid lipid NPs and nano-emulsions which consists of inner liquid core surrounded by an outer polymeric membrane called nanocapsule. Nanocapsules possess a polymeric membrane with a liquid nucleus, in which the active compounds of EOs is confined to a cavity [188]. Similarly, nanospheres are solid colloidal fragments in which bioactive components of EOs are diffuse, captured, encapsulated, or adsorbed into the polymer matrix [189]. Nano-encapsulation increases the physical stability of EOs, enhance bioactivity, reduce toxicity, and protect it from environmental interactions such as moisture, light, oxygen, pH, and controlling the release of EO. However, only nano-encapsulation, nano-emulsion, and monometallic nanoparticles with EOs has reported so far with limited data. Thus, EOs with their antimicrobial activity when blended with other potent antimicrobial agents like bi- and tri-metallic nanoparticles might be a probable source of alternative antimicrobial agents to combat multidrug resistant pathogens. 

**Figure 3:**
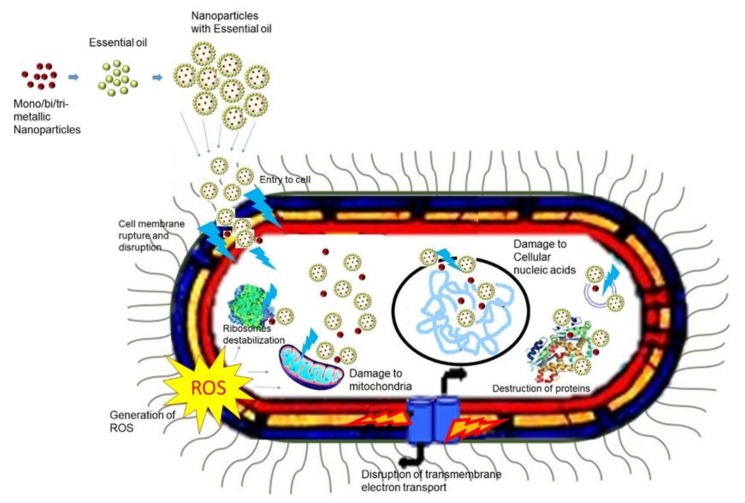
Proposed antibacterial mechanisms of mono, bi-, tri-metallic NPs with EOs. Combination of NPs and EOs can attack bacteria cell through multiple mechanisms; direct interaction with cell membrane by generating metal ions, disruption of cell membrane, protein dysfunction, DNA damage, inhibition of the electron transport chain, and the regulation of bacterial metabolic processes.

The constituents of EOs along with bi- and tri-metallic NPs with their diverse sizes, shapes, high surface-to-volume ratios, physical and chemical stability, and degree of selectivity can unlock the cell membrane channels, thus opening the passage of EOs/multimetallic to reach their target sites. These metal NPs or nanoemulsions/nanoencapsulations enclosing EOs can be adhered via electrostatic, hydrogen bonding, and covalent interactions to produce antimicrobial packaging systems. Once NPs adhere to cell walls, they directly effects toxicity due to larger concentration of NPs release more ions and distributed in the environment surrounding the bacterial cell wall. The larger concentration of generated ions disrupt cell membrane and ROS production and further helps to penetrate the cells [190]. When NPs enter inside the cell along with EOs cause damages in the structure of cell membranes, protein dysfunction causes oxidative stress and DNA damage as shown in the schematic representation in Figure 3. 

There are two possible ways to combat multidrug resistant pathogens along with EOs either by nano-encapsulation method or combination with bi- and tri-metallic NPs with EOs. Recently, Wu et al., prepared chitosan NPs embedded with *Torreya grandis* aril EOs and studied their antibacterial properties against *S. aureus* found stronger antibacterial activities than chitosan NPs alone [191]. Synergistic antibacterial activity of silver NPs with EOs of *Kelussia odoratissima* and *Teucrium polium* investigated their enhanced effect against multidrug-resistant bacteria [192]. Chitosan NPs encapsulated with *Cymbopogon martinii* EOs shows efficient and enhanced antifungal and antimycotoxin activities against *Fusarium graminearum* [193]. Similarly, silver NPs with eucalyptus EO showed the synergistic effect on the growth inhibition of *E. coli*, MRSA, *S. enteric*, and *B. subtilis* [194] and *Nigella sativa* EO coated with gold nanoparticles effectively controlled the growth and biofilm formation of S. aureus [195]. Whereas, rosemary and oregano EOs with silver and ZnO nanoparticles incorporated into pullulan films were effective against *S. aureus*, *L. monocytogenes*, *E. coli*, and *S. Typhimurium* [196].

## 10. Synergistic Antimicrobial Activity of Essential Oils

The development of resistance to different antimicrobial agents by bacteria, fungi, viruses, and parasites is a great challenge to the effective treatment of infectious diseases, and hence there is a need for more intensive, new, and novel antimicrobials. Some plant extracts and EOs have been used as medicine for the treatment of infectious diseases traditionally. However, these plant-based medicines are not effective for severe systemic infections due to the absence of clinically applicable pharmaceutical forms. Subsequently, many antibiotics have been developed as synthetic antimicrobial agents, but these drugs are complicated by their high toxicity, low tolerability, and ineffectiveness against new emerging microbes. One probable way to enhance the range and scope of current antimicrobial therapy is the use of a combination of antimicrobials. 

The combination of antimicrobial agents such as essential/volatile oils, plant extract/EOs, plant extracts/antibiotics, EOs/EOs, EOs/antibiotics, plant extract/plant extract, EOs/monometallic NPs, and phytochemical/antibiotics [16] has confirmed the significant effects of antimicrobial activities. In addition, nanoencapsulation of many EOs and their antimicrobial activities have also been discussed in the previous section. Synergy can be assessed by combining two antimicrobial compounds and conducting antibacterial activity, whereby the sum of the antibacterial activities is greater than antibacterial activity of the individual components due to several substances, which improves solubility. According to Hossain et al. [197], combination of eight EOs of plants showed enhanced antimicrobial activity against *A. niger*, *P. chrysogenum*, *A. flavus*, and *A. parasiticus* when compared to the antimicrobial activity of a single EO. Similarly, in a study conducted by Knezevic et al. [198], it was reported that the combination of EOs of *Eucalyptus camaldulensis* with conventional antibiotics such as gentamycin, ciprofloxacin, and polymyxin B showed synergistic antibacterial effect against *Acinetobacter baumannii.*

Very few researchers have studied the synergistic antimicrobial effects of metal and metal oxide NPs (monometallic NPs) along with EOs and conventional antibiotics [199,200]. Scandorieiro et al. [201] reported that the combination of silver NPs with *Origanum vulgare* EO resulted in synergistic antimicrobial activities against *E. coli*, *A. baumannii*, and *S. aureus*. Similarly, hydroxyapatite NPs with peppermint EO against *S. aureus*, *E. faecium*, *E. coli*, *P. aeruginosa*, and *C. parapsilosis* [202], olive EO with lipid nanoparticles against *P. pyogenes* and *S. aureus* [203], *Siparuna guianensis* EO with chitosan NPs against *A. aegypti* [187] showed enhanced antimicrobial effects. In addition, rosemary and oregano EOs, with silver and zinc oxide NPs incorporated into pullulan films were effective against pathogenic microorganisms such as *S. aureus*, *L. monocytogenes*, *E. coli*, and *S. typhimurium* [196], cinnamon EO with chitosan NPs against *E. coli* and *S. aureus* [204] also had synergistic antimicrobial activities.

## 11. Challenges and Future Directions

EOs have great potential for the promotion of health and preventing and treating infectious diseases. However, EOs have some drawbacks due to their low solubility, stability, and high volatility in medicinal applications. Hence, the encapsulation of EOs increases their solubility and stability, and maintains controlled release, which makes them more bioavailable; protects them from air, humidity, light; and lead to volatilization, increasing their biological activities. To date, there are limited studies on synergistic antimicrobial effects of metal and metal oxide NPs along with EOs. Bi-and tri-metallic NPs have recently attracted more attention because of their unique catalytic, optical, electronic, and magnetic properties [19], and their utilization is associated with their well-defined properties when compared to their monometallic counterparts [205,206]. 

Bi- and tri-metallic NPs are formed by the combination of two or more different metals or materials into a single system, and they exhibit special properties which are not observed in the case of the monometallic forms [207]. Moreover, these bi- and tri- metallic NPs are used as catalysts for many organic transformations [23] with excellent selectivity and activity. In addition, altering the shape, size, and/or surface chemistry of NPs as core–shell, heterodimer, and nano-alloys [23,28,29] enhance their catalytic performance. As discussed earlier in the previous section, several studies on of the nanoencapsulated EOs and metal and metal oxide NPs have demonstrated potential antimicrobial activities and synergistic effects, which have already been documented. 

The present study acknowledged more findings on in vitro studies of EOs, and nanoencapsulation of EOs, however, more efforts are required to conduct studies on the synergistic effects of bi- and tri-metallic NPs along with different EOs. Tuning into different size, shape, and/or surface chemistry of NPs allows their functionalities to be more enhanced for better applications. Furthermore, the use of multi-component nanoparticles like bi- and tri-metallic NPs comprised of three or more metals have also intervened as hybrid materials that can upgrade optical and magnetic properties. However, the inertness of these materials with respect to the environment, health, and safety concerns must be considered due to their potential hazards and toxicity. Thus, EOs along with mono, bi-, and tri-metallic NPs might be a prospective source of alternative antimicrobial agents, and may play an important synergistic role in the discovery of new drugs for the treatment of a wide range of pathogenic infections in the near future. Finally, we will continue to see creative advances in the synthesis of these unique dual-component and multi-component nanomaterials and their antimicrobial activities along with EOs individually or in combinations.

## 12. Conclusions

The increasing number of clinical complications related to multidrug resistant microorganisms has inspired researchers to focus their interest on alternative antimicrobial agents, which are an appropriate solution to treating infections that are more serious to human health. There are several types of EOs, which have individual components with diverse bioactivities including antimicrobial potential. However, EOs have certain limitations, including low solubility and stability, and currently they have reduced clinical applications owing to the development of resistance. Nanotechnology is expected to have a significant impact on the therapeutics of antimicrobial agents at the nanoscale level to develop nanomedicines. As the size of a particle decreases, the specific surface area, reactivity, and bioavailability increases, allowing greater interaction with the surrounding environment that enhances the antimicrobial effects. Indeed, nanoparticles are able to solve the major inconvenience of EO components by increasing the chemical stability in the presence of moisture, air, light, and high temperatures, factors which can lead to the rapid evaporation and to the degradation of the active components. Encapsulation simultaneously increases the antimicrobial potency of EOs by controlled or sustained release and facilitating close interaction with the microorganisms.

Metal and metal oxide NPs like Ag, Au, Ga, FeO, ZnO, TiO, MgO, and CuO act as promising antimicrobial agents. Bi-metallic or multimetallic nanostructures such as hybrid, core–shell, or alloy structures like FePd, AuPd, CuPd, PtPd, AuFeAg, and FeAgPt enhance antimicrobial activities and catalytic performance. For this reason, it is necessary to find an alternative method to combat multidrug resistance pathogens by using EO encapsulation with suitable metal or metal oxide NPs or combination of EOs with different bi- and tri-metallic NPs. The present study revealed more information on the extraction methods, chemical composition, nano-encapsulation, and antimicrobial activities of EOs and antimicrobial potential of some metal and metal oxide NPs. The future biotechnological perspective to stabilize EOs using nanostructured materials might establish a valuable prerequisite for obtaining functionalized materials with modified surface, inhibitory effects and ability to adhere on the microorganisms for targeting and/or controlled release. However, more research is required to explore the mechanism of individual essential oil components along with bi- and tri-metallic NPs with an initiation in systematically conduct experiments on the synergistic mechanisms among different components. Therefore, new or alternative strategies for EOs with bi- and tri-metallic NPs and synergistic studies can provide an interesting platform in the future to combat multidrug resistance pathogens with greater activity. In addition, it is necessary to control the toxicity, risk assessment, and safety aspects, and to avoid the passage of such nanomaterials into the human body. 

## Figures and Tables

**Table 1:** Various extraction methods for different essential oils

Common Name	Scientific Name	Plant Parts	Extraction Method	References
Rosemary	*Rosmarinus officinalis*	Leaves	Hydrodistillation	[52]
Pu-erh ripe tea	*Camellia sinensis*	Leaves	Soxhlet extraction	[53]
Chokeberry	*Aronia melanocarpa*	Fruits	Maceration	[54]
Lemon	*Citrus limon*	Fruits	Cold pressing	[55]
Lavandin	*Lavandula angustifolia*	Flowers	Supercritical fluid	[56]
Lavender	*Lavandula angustifolia*	Flowers	Ultrasound-assisted	[57]
Black cumin	*Nigella sativa*	Seeds	Microwave-assisted	[58]
Oregano	*Origanum vulgare*	Leaves	Infusion and decoction	[59]

**Table 2:** Major chemical composition of various EOs and their antimicrobial activity against pathogenic microorganisms

Plant Source	Plant Part	Major Chemical Compounds	Microorganisms	References
*Fortunella margarita*	Leaves	Gurjunene, eudesmol, muurolene	*B. subtilis, S. aureus, Sarcina luta, S. faecalis, E. coli, K. pneumonia, P. aeruginosa*	[88,89]
*Eremanthus erythropapps*	Leaves	Germacrene D, p-cymene, 𝛾-terpinene	*S. epidermidis*	[90]
*Euphrasia rostkoviana*	Commercial EOs	n-Hexadecanoic acid, thymol, myristic acid, linalool	*E. faecalis, E. coli, K.* *pneumoniae, S. aureus, S. epidermidis, P. aeruginosa*	[91]
*Pogostemon cablin*	Leaves	Patchoulol	*H. pylori*	[92,93]
*Plectranthus neochilus*	Leaves	𝛼-Pinene, trans-caryophyllene	*S. mutans*	[94,95]
*Ocimum basilicum*	Arial parts	Linalool, methyl chavicol	*M. flavus*	[96,97]
*Salvia sclarea*	Arial parts	Linalool, linalyl acetate	*E. coli, S. aureus, B. subtilis, S. typhimurium, K. pneumonia, P. Aeruginosa, B. pumilus*	[98]
*Thymus kotschyanus*	Arial part	Carvacrol, 1,8 cineole, thymol, borneol	*S. aureus, S. epidermidis, B.* *cereus, E. coli*	[99,100]
*Glechon marifolia*	Leaves	𝛽-Caryophyllene, bicyclogermacrene	herpes simplex virus type 1	[101]
*Myrtus communis*	Leaves	α-Pinene, 1,8-cineole	*C. albicans, A. flavus*	[102,103]
*Origanum vulgare*	Leaves	Carvacrol	*T. tonsurans, T. violaceum, T. floccosum, T. mentagrophytes*	[104,105]
*Syzygium aromaticum*	Leaves	Eugenol, eugenylacetate	*C. albicans, Candida* spp.	[106,107]
*Pelargonium graveolens*	Leaves	Citronellol, geraniol	*C. tropicalis*	[108,109]
*Trachyspermum ammi*	Leaves	Thymol, 𝛼-pinene,	Japanese encephalitis virus	[110,111]
*Lepechinia salviifolia*	Leaves	Germacrene D	Herpes simplex virus type 1	[112]
*Lavandula x intermedia*	EOs	Linalool, camphor and 1,8-cineole	*L. monocytogenes*	[113,114]
*Thymus vulgaris*	Leaves	Carvacrol	*M. furfur*	[115]
*Mentha piperita* L.	Leaves	Menthol	*C. albicans, C. tropicalis, P. anomala and S. cerevisiae*	[116,117]
*Melaleuca cajuputi*	Leaves	1,8-Cineole, linalool, terpinen-4-ol	*Aspergillus spp. A. niger*	[118]
*Cinnamomum zeylanicum*	Bark	Carvacrol	*Borrelia Burgdorferi*	[119,120]
*Eugenia caryophyllata*	Clove buds	Eugenol, 𝛽-caryophyllene	*S. aureus*.	[121]
*E. loxophleba*	Leaves	1,8 Cineole	*S. aureus and E. coli*	[122]
*Salvia officinalis* L.	Leaves	1,8-Cineole, α-thujone, camphor	*B. subtillis and S. epidermidis*	[123,124]
*Melaleuca alternifolia*	Leaves	Terpinen-4-ol	*C. albicans*	[125,126]
*Coriandrum sativum* L.	Fruits	Linalool	*E. coli B. bronchiseptica*	[127,128]
*B. dracunculifolia*	Leaves, flowers	Spathulenol, nerolidol,	*S. aureus, B. cereus, and* *P. aeruginosa.*	[60]
*Ocimum basilicum*	Arial parts	Linalool	*C. albicans, S. aureus*	[129]
*Rosmarinus officinalis*	Leaves	1,8-Cineole, camphor	*C. perfringens*	[130]
*Epilobium parviflorum*	Arial parts	Oenothein B, myricitrin	*E. fecalis, S.aureus*	[131]

**Table 3:** Antimicrobial activity of mono, bi-, and tri-metallic and metal oxide nanoparticles, specifically highlighting size, shape, bacterial strains tested, and mode of action

NPs	Size and Shape	Bacteria Pathogens	Mode of Action	Ref.
Ag	15 nm, triangular	*P. aeruginosa* and *E. coli*	Deactivation of enzymes and cellular proteins	[158]
	23 nm,	*S. typhimurium*	Interaction of NPs with membrane proteins	[159]
	20 nm, triangular	*E. coli* and *S. aureus*	Destruction of outer and inner membrane	[160]
	7.1 nm, spherical	*E. coli* and *P. aeruginosa*	Permeabilized membrane	[161]
	25 nm, spherical	*S. aureus,* and *E. coli*	Structural changes in the cell wall and nuclear membrane	[162]
Au	10 nm, spherical	*S. aureus* and *P. aeruginosa*	Disruption of cell membrane	[163]
	20 nm, spherical	*S. pneumoniae*	Disruption of cell membrane	[164]
	1-3 nm, spherical	*E. coli, P. aeruginosa, S. epidermidis* and *B. subtilis*	Interaction between NPs and bacteria could induce a metabolic imbalance	[165]
	50 nm, spherical	*S. oneidensis*	Interaction of NPs with membrane proteins	[166]
Ga	305 nm, rod	*M. smegmatis* and *HIV*	Disruption of cell membrane	[167]
Ag/Au	30 nm, triangular,	*B. subtilis, E. coli, S. typhi,* and *S. aureus*	Interaction between NPs and vital components leads to enzyme inactivation	[168]
Cu/Pt	30 nm, spherical	*E. coli, S. aureus, P. aeruginosa,* and *C. albicans*	Permeabilized membrane	[169]
Al/Ag	200 nm, spherical	*E. coli,* and *S. aureus*	Adsorption and inactivation of bacterial strains	[170]
Fe/Cu	68 and 82 nm, spherical	*S. aureus,* and *P. aeruginosa*	Structural changes in the cell wall and nuclear membrane	[171]
Cu/Cr/Ni	100 and 200 nm, plate	*E. coli* and *S. aureus*	Rupture of the membrane and denaturation of bacterial proteins	[172]
Cu/Zn/Fe	42 nm, spherical	*E. coli* and *E. faecalis.*	Disruption of cell membrane	[173]
Au/Pt/Ag	20-40 nm, spherical, triangle, ellipsoidal	*E. coli, S. typhi, Klebsiella, E. coli and,* and *E. faecalis*	Interaction with the cell components such as DNA and enzymes	[174]
ZnO	20 nm, spherical	*S. typhimurium, and S. aureus*	Cell wall damage	[175]
CuO	198 nm,	*B. cereus, P. mirabilis and A. caviae*	Loss of membrane integrity and increased permeability	[176]
MgO	24 nm,	*S. epidermidis*	Disruption of cell membrane	[177]
TiO_2_	50 nm	*P. fluorescens and E. coli*	Destruction of membrane, DNA and proteins	[178]
